# Actual Clinical Practice Assessment: A Rapid and Easy-to-Use Tool for Evaluating Cognitive Decline Equivalent to Dementia

**DOI:** 10.7759/cureus.58781

**Published:** 2024-04-22

**Authors:** Takayuki Asano, Asako Yasuda, Setsuo Kinoshita, Jun Nomoto, Takahiro Kato, Chihiro Suzuki, Han Suzuki, Toru Kinoshita, Masahiro Shigeta, Akira Homma

**Affiliations:** 1 Research and Development, Nippontect Systems Company Limited, Tokyo, JPN; 2 Brain Neurosurgery, Hamato Neurosurgery Clinic, Kanagawa, JPN; 3 Brain Neurosurgery, Soubudai Neurosurgical Clinic, Kanagawa, JPN; 4 Brain Neurosurgery, Neuroscience Center of Suzuki Neurosurgical Clinic, Saitama, JPN; 5 Psychiatry, Nozomi Memory Clinic, Tokyo, JPN; 6 Department of Psychiatry, The Jikei University School of Medicine, Tokyo, JPN; 7 Psychiatry, Otafuku Memory Clinic, Ibaraki, JPN

**Keywords:** automated classification, machine learning, speech features, dementia, digital cognitive assessment

## Abstract

Background

Screening tests reveal the early signs of cognitive decline, enabling better self-care and preparation for the future. We developed and evaluated the accuracy of a rapid (20 s) and easy-to-use tool called ONSEI, assessing the cognitive decline equivalent to dementia in actual clinical practice by correlating clinical diagnoses with the ONSEI classification.

Methods

In this retrospective observational study, data were collected from individuals who visited three neurosurgical clinics in neighboring prefectures of Tokyo, Japan. ONSEI analysis was performed using a smartphone or tablet. The tool adopts a machine-learning algorithm using the speaker’s age, time-orientation task score, and acoustic features of spoken responses to that task. Significant differences in accuracy, sensitivity, and specificity were evaluated by Fisher’s exact test.

Results

The overall classification accuracy of ONSEI was 98.1% (p<0.001). The sensitivity and specificity were 97.3% (p<0.001) and 98.5% (p<0.001), respectively. The proportion of correct classifications was consistent across different age groups.

Conclusion

ONSEI showed high classification accuracy for dementia in cognitively normal individuals in actual clinical practice, regardless of the facility at which the tests were conducted or the age of the participants. Thus, ONSEI can be useful for dementia screening and self-care.

## Introduction

Currently, nearly 10 million people are diagnosed with dementia recently each year worldwide [1]. Screening tests can alert individuals to early signs of cognitive decline, enabling better self-care and preparedness for the future. Early detection also reportedly promotes early intervention and treatment, thereby delaying cognitive decline and reducing the risk of developing dementia [2,3]. Furthermore, the anti-amyloid-beta antibody approved by the U.S. Food and Drug Administration for the treatment of Alzheimer’s disease underscores the need for early diagnosis [4]. Therefore, simple and efficient cognitive assessment tools are needed that can be used in a wide range of settings, such as primary health care centers, community settings, or homes.

Advances in digital technology have led to attempts to create easy and convenient dementia detection tools using digital devices. In addition to mobile versions of existing articles or computerized neuropsychological tests, new types of tablet‐ and smartphone-based cognitive assessment tools are developed using digital technology such as spoken language analysis, automated language processing, eye tracking, and virtual reality [5,6]. For example, the Cognitive Assessment for Dementia, iPad version (CADi), consists of 10 items of neuropsychological tests which take approximately 10 minutes to complete [7]. Oyama et al. developed a brief and practical cognitive assessment tool using eye-tracking technology with short-task movies and pictures to assess cognitive function. The eye tracking-based cognitive scores, which were highly correlated with traditional neuropsychological tests, including the Mini-Mental State Examination, the Alzheimer’s Disease Assessment Scale-cognitive subscale, and the Frontal Assessment Battery, were obtained in a short period of time (178 s) [8].

Older adults still face challenges due to the digital divide, including technology anxiety and limited digital literacy. However, most cognitive assessment tools are touch-based, which require operation by individual participants [5,6]. This is predicted to result in lower acceptance and performance among older adults. Therefore, digital cognitive assessment tools need to be easier to use and reduce the burden on users to be accepted by older adults. For example, a digital tool that can detect the cognitive decline equivalent to dementia through speech alone in a rapid and easy-to-use manner appears to be a promising solution and is accepted by people of all ages.

In our previous study [9], we found that machine-learning classifiers using the scores of the time-orientation task or delayed recall task and acoustic features derived from the responses to the respective tasks were effective in distinguishing between cognitively normal and mild Alzheimer's disease (AD) in the Hasegawa's Dementia Scale-Revised. Specifically, a gradient boosting classifier that utilized the time-orientation score and participant age showed an accuracy of 81.9%. Classifiers that incorporated the modulation spectrum or emobase as acoustic features achieved an accuracy of 86.1% and 88.9%, respectively. These accuracies are comparable to those of delayed recall, which is considered to have a high discrimination performance. Therefore, a classifier that can be executed through a time-orientation task in a few seconds may be highly beneficial in practical applications.

This study aimed to develop a rapid and easy-to-use application to assess the cognitive decline equivalent to dementia. Based on our previous research [9], we developed ONSEI (meaning 'voice' in Japanese). Furthermore, we investigated the degree of coincidence between the clinical diagnosis and classification by ONSEI to evaluate the accuracy of the test in actual clinical practice. Our study showed that ONSEI demonstrated high classification accuracy versus clinical diagnosis accuracy.

## Materials and methods

ONSEI development

We developed ONSEI, a native application for personal use, and a web application for medical institutions. ONSEI assesses cognitive decline equivalent to dementia based on the speaker’s age, score of a time-oriented task, and acoustic features of the spoken responses to the task. The test takes approximately 20 seconds from implementation to the automatic output of the classification. The web application has an additional feature that allows registration of the diagnostic name of the speaker. In this application, the speaker was asked the date and day of the current week in the Western calendar to respond after registering their date of birth. 

The speaker’s speech is recorded and saved in the Resource Interchange File Format (RIFF WAV) with a 16 kHz sampling rate. The audio is also power-normalized to ensure consistent amplitude. The recorded data are then converted to text using Google Speech-to-Text and automatically scored from 0 to 4 points based on the accuracy of the answer. 

To construct a machine learning algorithm, we used the modulation spectrum as an acoustic feature. The modulation spectrum is defined as the log power spectrum of the acoustic feature time series and serves as a long-term audio characteristic [10]. It follows Fourier's law and captures the temporal changes present in the acoustic feature time series. Although the modulation spectrum can be derived from the temporal variation of any short-term acoustic feature, we specifically computed and used 20 lower-order components of the modulation spectrum derived from Mel-frequency cepstral coefficients. ONSEI was implemented using a gradient-boosting classifier that utilized the speaker’s age, time-orientation task score, and acoustic features.

Study design and data collection

This was a retrospective observational study that used data obtained as part of medical treatment in actual clinical practice. Data were collected from individuals who visited three neurosurgical clinics in neighboring prefectures of Tokyo, Japan, and ONSEI was implemented between November 6, 2019, and November 30, 2022. Analysis via ONSEI was performed using a smartphone or tablet. Each speaker’s date of birth and diagnosis was registered by the physicians onto the tool. The diagnosis by a physician was registered as cognitively normal or dementia according to the fifth edition of the Diagnostic and Statistical Manual of Mental Disorders (DSM-5) criteria for dementia. The data collected in this study included all types of dementia as they were acquired in actual clinical practice. Ninety-six individuals with missing data and 10 individuals under 20 years of age or over 100 years of age were excluded. There were no refusals to participate, and data from 1586 participants were included in the analysis.

The Declaration of Helsinki was followed, and the study complied with the research protocol and the Ethical Guidelines for Medical and Health Research Involving Human Subjects (partially revised on March 10, 2022). As this was a retrospective observational study, the opportunity to refuse to participate was provided on the bulletin board of the clinic. In accordance with the Personal Information Protection Law, adequate management steps were taken to ensure the security of the personal information of the participants included in this study.

Statistical analysis

Statistical analyses were performed using R software (v.4.2.2; R Foundation for Statistical Computing, Vienna, Austria). Fisher’s exact test was used to evaluate the classification accuracy of ONSEI, and the significance level was set at p<0.05.

## Results

Among the 1586 participants in this study, 1097 (69.2%) belonged to the cognitively normal group and 489 (30.8%) belonged to the dementia group. The number and average age of participants in each group are shown in Table 1.

**Table 1:** Demographics SD: Standard deviation

	Total		Facility A		Facility B		Facility C	
	Dementia	Cognitively normal	Dementia	Cognitively normal	Dementia	Cognitively normal	Dementia	Cognitively normal
n	489	1097	284	498	187	557	18	42
Age (mean ± SD)	79.9 ± 8.7	73.9 ± 13.1	78.6 ± 9.7	70.6 ± 13.8	82.0 ± 6.5	78.4 ± 9.2	78.4 ± 8.8	54.3 ± 17.7

Table 2 displays the accuracy, sensitivity, and specificity for each facility as well as the aggregated results for all facilities. The overall classification accuracy of ONSEI, representing the concordance rate with the clinical diagnoses, was 98.1%. The sensitivity and specificity were 97.3% and 98.5%, respectively. Fisher’s exact test revealed that ONSEI exhibited high classification accuracy versus clinical diagnosis accuracy (p<0.001), and the high classification accuracy of ONSEI was also observed in each facility (p<0.001). Statistical analysis of the results between the facilities showed significant differences in accuracy (p = 0.015) and sensitivity (p = 0.010).

**Table 2:** Accuracy, sensitivity, and specificity of ONSEI The accuracy, sensitivity, and specificity in total and per facility are shown. Values in parentheses indicate the number of participants correctly classified out of the respective total number.

	Accuracy	Sensitivity	Specificity
Total	98.1% (1556/1586)	97.3% (476/489)	98.5% (1080/1097)
Facility A	97.8% (765/782)	98.2% (279/284)	97.6% (486/498)
Facility B	98.8% (735/744)	97.3% (182/187)	99.3% (553/557)
Facility C	93.3% (56/60)	83.3% (15/18)	97.6% (41/42)

**Figure 1:**
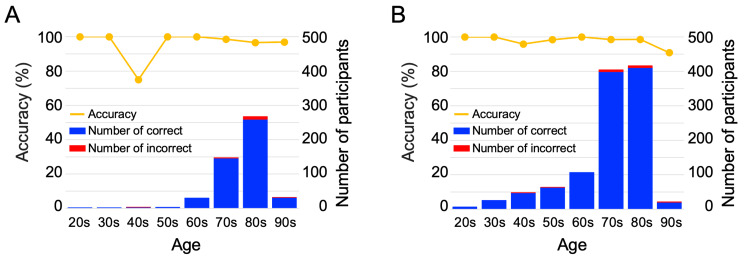
The accuracy and number of participants in each generation (A) Dementia group. (B) Cognitively normal group. The yellow line indicates the percentage of correct classifications. Blue and red bars indicate the number of correctly and incorrectly classified participants, respectively.

To determine whether the classification accuracy of ONSEI differed according to the participant’s age, we calculated the percentage of correct answers based on age for each group (Figure 1). Although the age distribution was skewed because the data included many individuals in their 70s and 80s, the percentage of correct answers for each age group was over 90.0%, except for those in their 40s in the dementia group, who had a correct answering rate of 75.0% owing to a small population size (one individual misclassified out of four).

## Discussion

In this study, we developed and evaluated the accuracy of ONSEI to assess the cognitive decline equivalent to dementia in actual clinical practice by examining the correlation between clinical diagnoses and the ONSEI classification. ONSEI demonstrated high classification accuracy, as measured by its concordance rate with the clinical diagnoses. These findings validate ONSEI as a rapid, voice-dependent, and automated cognitive assessment tool compatible with smartphones or tablets. ONSEI is an easy and convenient tool for detecting dementia even for older adults. ONSEI might overcome the shortcomings of low acceptance and performance in touch-based tools. This tool may be particularly useful in situations in which specialized services are unavailable because of a shortage of specialists in the neighborhood, difficulty in leaving one’s home, or hesitancy in consulting a physician for diagnosis.

Statistical analysis of the results showed significant differences in accuracy and sensitivity between facilities (p<0.05). One reason for this discrepancy could be related to the differences in the participant population at each facility and between regions. Additionally, the number of participants at one of the facilities was small, which may have contributed to the high rate of misclassification at that facility. Nevertheless, ONSEI exhibited high classification accuracy at all facilities (p<0.001), suggesting that it is suitable for use at any facility.

Although most of the currently available digital cognitive assessments are touchscreen-based, two studies have reported successful screening by voice, using tests that require no operation by participants. One study found that using a convolutional neural network model and voice recordings from neuropsychological tests could detect dementia in normal individuals with up to 74.0% accuracy [11]. Another study reported that 3- to 5-minute speech recordings taken from a free conversation could be used to classify dementia versus non-dementia with an accuracy of 90.0% using a machine learning tool [12]. Thus, ONSEI has the potential to achieve detection accuracy as good as or even better than that obtained in previous studies, with a short test time of 20 seconds.

In recent years, there has been a trend toward early diagnosis of Alzheimer’s disease before it crosses the threshold of dementia [13]. This “timely diagnosis” potentially offers the opportunity for early intervention, better management of symptoms, time to seek support, crisis avoidance, cost savings, and prolonged periods of mild conditions [3]. The use of a cognitive screening test can also facilitate early diagnosis, which in turn helps older adults with dementia and their families develop short-term coping and long-term care plans to provide a more comfortable way of life [14].

The present study showed that ONSEI provides high classification accuracy in actual clinical practice. ONSEI can be self-administered in a short time, which makes it more flexible for any time and anywhere assessment, making it feasible for regular monitoring of cognitive status. ONSEI can be used by simply installing an application on a smartphone or tablet. Because of its convenience, the commercial version of ONSEI has already been introduced in several municipalities and companies in Japan. We believe that ONSEI, in addition to serving as a useful tool for assessing cognitive decline equivalent to dementia in primary healthcare and community settings, will be utilized as a high-validity self-care tool in the future.

The limitations of this study include a lack of information on the participants apart from age and diagnosis (cognitively normal or with dementia). Background information on parameters such as sex and years of education, as well as disease information such as the type and severity of dementia or cognitive test scores, was also not available. Therefore, we were unable to verify the impact of these factors on ONSEI classification. In addition, the number of participants at facility C was small. Differences in the number of participants and age distributions among the facilities may affect the results of the classification accuracy. Moreover, the data used in this study were limited to the neighboring prefectures of Tokyo, where most residents speak standard Japanese. However, because the input of spoken words in ONSEI is short, the effect of dialect is considered small. Further research should address these limitations.

## Conclusions

The findings of our study suggest that ONSEI is a promising tool for the assessment of cognitive decline equivalent to dementia. The test can be self-administered in a short time. Thus, ONSEI is a useful tool for detecting dementia in primary health care and community settings and can be further utilized as a high-validity self-care tool. In the future, we will investigate the relationship between ONSEI outputs and speakers’ sex, years of education, disease information, and dialect, thereby further demonstrating the extensive usefulness of ONSEI.
